# Neutrophil/Lymphocyte and Platelet/Lymphocyte Ratio in Preterm Premature Rupture of Membranes

**DOI:** 10.7759/cureus.38664

**Published:** 2023-05-07

**Authors:** Alev Esercan, Ismail Demir

**Affiliations:** 1 Obstetrics and Gynecology, Şanlıurfa Education and Research Hospital, Şanlıurfa, TUR

**Keywords:** preterm birth, preterm premature rupture of membranes, platelet lymphocyte ratio, neutrophil-lymphocyte ratio, inflammation

## Abstract

Aim: This study aimed to investigate maternal hematological laboratory parameters of pregnancies complicated with preterm premature rupture of membranes (PPROM) compared to mothers of healthy-term infants (control group).

Materials and methods: This case-control study included 158 mothers who were divided into PPROM (n=67) and control (n=91) groups, respectively. Laboratory parameters of platelet, lymphocyte, and neutrophil counts, neutrophil/lymphocyte ratio (NLR), and platelet/lymphocyte ratio (PLR) were recorded at the time of diagnosis for the PPROM group and at the time of hospital admission for birth for the control group.

Results: NLR was significantly higher in the PPROM group than in the control group (p=0.001). The cutoff value of NLR to predict PPROM was 6.73 (AUC=0.671, 95% confidence interval=0.58-0.75, p=0.000).

Conclusion: A cheap and routine NLR blood test can be used to predict PPROM before labor.

## Introduction

Preterm premature rupture of membranes (PPROM) is the rupture of the membranes before the 37th gestational week, with a prevalence of approximately 3% in pregnancies (0.5% for <27 gestational weeks, 1% for 27-34 gestational weeks, and 1% for 34-37 gestational weeks). The pathophysiology of PPROM is not clear. Infection, inflammation, and bleeding are known etiologies, but the initiating event is usually non-identifiable [1]. Labor induction is a common practice in pregnancies with PPROM at ≥34 weeks of gestation unless there is spontaneous labor or the presence of chorioamnionitis, abruptio placentae, or cord prolapse. Although hospital deliveries and the quality of pregnancy follow-up have increased recently, the worse fetal outcome of preterm birth due to PPROM can not be solved.

In our study, we aimed to compare maternal hematological laboratory parameters of PPROM patients with those of mothers of healthy-term infants and investigate whether there was actual inflammation in mothers of infants with PPROM. We also compared the inflammatory parameters of a study group with those of a control group to determine whether they had predictive value.

## Materials and methods

Pregnant women who applied to Şanlıurfa Education and Research Hospital between January 2018 and January 2022 with the diagnosis of PPROM between 22nd and 34th gestational weeks were included in the study group, and pregnant women who delivered after at least 37 weeks of gestation without PPROM were included in the study as a control group. The hematological analysis levels of the patients with PPROM at their first hospitalization and those of the control group at the time of delivery were recorded. All selected patients were in the normal range for c-reactive proteins.

The exclusion criteria of the study were multiple pregnancies; pregnant women with preterm birth risks such as cerclage, fetal anomalies, and cigarette smoking; PPROMs without birth registration; hematological disorders; and pregnant women with active signs of infection. Both groups were assessed for risk factors such as gestational diabetes, in vitro fertilization (IVF) pregnancies, and gestational hypertension. Additionally, laboratory parameters, including platelet count, neutrophil count, lymphocyte count, neutrophil/lymphocyte ratio (NLR), and platelet/lymphocyte ratio (PLR), were recorded in both groups.

The data were evaluated using the SPSS 26.0 statistics program (IBM Corp, Armonk, NY), and percentages were calculated for the mean, standard deviations, and categorical data. Student’s t-test was used to compare means between groups, the chi-squared test was used for categorical data, and correlation analysis was performed for the significant means. Receiver operating characteristic curve (ROC) curves were drawn. In this study, a p-value <0.05 was considered statistically significant.

Permission for the study was obtained from the Ethics Committee of Harran University, Şanlıurfa, Turkey (Date: 23 May 2022, Decision No.: 22.10.03). All procedures followed the ethical rules and the principles of the Declaration of Helsinki.

## Results

A total of 158 patients, 67 PPROM (group 1) and 91 control (group 2), were included in this study. The mean maternal age in PPROM and control groups was 30.17±7.24 and 24.47±4.05 years, respectively. Maternal age in the PPROM group was greater than the control group and the difference was statistically significant (p: 0.01). The mean gravida in the PPROM and control groups was 4.94±3.06 and 3.08±1.44, and the mean birth weight was 1756.34±697.14 and 3191.51±380.30 grams. Gravida of the PPROM group was higher than that of the control group and the differences were statistically significant from the control group (p: 0.00). There was a statistically significant difference according to risk groups in the PPROM group rather than the control group (p: 0.002). 11.9% of patients in the PPROM group (especially the most risky was gestational diabetes) and 1.1% of patients (gestational hypertension) had risk in the control group. The vaginal birth rate was 65.9% in the control and 32.8% in the PPROM group (p: 0.00).

Although there was no statistically significant difference between PPROM’s and the control group’s hematological parameters according to gestational week (Table 1), the mean of the total PPROM group’s NLR values was statistically different than the control group (both p <0.001). PLR values in the PPROM group at 25-30 gestational weeks were significantly higher than in the control group (p: 0.03).

**Table 1:** Hematological values of PPROM and control groups p: p-value of comparison between all PPROM groups and control group; p1: p-value of comparison between 22-24 wk PPROM-control; p2: p-value of comparison between 25-30 wk PPROM-control; p3: p-value of comparison between 31-34 wk PPROM-control; p<0.05 is statistically significant; *p<0.05.

Parameter	Mean±SD				p-Value			
	Group							
	PPROM			Control	p	p1	p2	p3
Gestational week	22-24 wk (n=19)	25-30 wk (n=34)	31-34 wk (n=14)					
Neutrophil count (10³/uL)	9.9±3.8	10.3±2.8	10.1±3.5	8.4±2.4	0.001	0.22	0.49	0.07
Lymphocyte count (10³/uL)	2.0±0.6	2.2±1.1	2.1±0.7	2.3±0.7	0.001	0.14	0.37	0.28
Platelet count (10³/uL)	248.5±59.7	272.5±64.2	249.3±52.9	244.7±70.8	0.589	0.41	0.23	0.41
Neutrophil/lymphocyte ratio (NLR)	5.9±4.8	6.1±5.3	6.1±5.5	3.8±1.9	<0.001	0.06	0.2	0.48
Platelet/lymphocyte ratio (PLR)	129.2±35.9	146.7±65.1	136.3±69.1	111.2±46.6	0.254	0.35	0.03*	0.15

**Figure 1:**
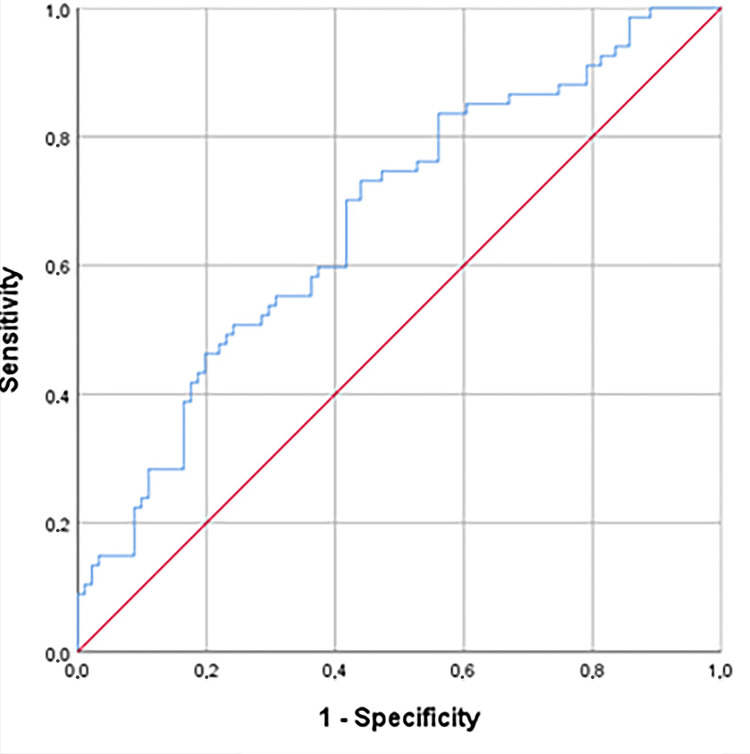
ROC curve of NLR in the PPROM group ROC: receiver operating characteristic; NLR: neutrophil/lymphocyte ratio; PPROM: preterm premature rupture of membranes.

NLR cutoff of 6.73 was obtained for PPROM occurrence (AUC=0.671; 95% confidence interval 0.58-0.75; p=0.000) (Figure 1, Table 2).

**Table 2:** Area under the curve for NLR in the PPROM group NLR: neutrophil/lymphocyte ratio; PPROM: preterm premature rupture of membranes.

Area Under the Curve					
Test Result Variable(s)	Area	Standard Error	Asymptotic Significance	Asymptotic 95% Confidence Interval	
				Lower Bound	Upper Bound
NLR	0.671	0.043	0.000	0.587	0.755

## Discussion

Our hospital is a tertiary center for obstetrics and newborns and is ranked first in Turkey in the total number of births (approximately 36,000 per year). In addition, many of the PPROM patients have their follow-up and treatment at the perinatology clinic in our hospital.

The NLR is calculated by dividing the neutrophil count by the lymphocyte count. In the literature, NLR, as an early biomarker of sepsis, correlates with prognosis, such as in the Acute Physiology and Chronic Health Evaluation II (APACHE II) score and the SOFA score [2,3]. It is worth noting that NLR increases rapidly following infection. Therefore, our study excludes patients with infection due to the possibility of increased NLR values. However, despite the exclusion, the PPROM group had higher NLRs.

As the presence of chorioamnionitis is considered the most important factor that determines prognosis in PPROM, many studies have focused on its early and noninvasive detection, including some on white blood cells (WBCs) and C-reactive protein (CRP). Sabogal et al. found that leukocytosis had low sensitivity (51%) and specificity (65%) in the diagnosis of chorioamnionitis [4]. There are also other tests, such as those of soluble intercellular adhesion molecule-1, interleukin-6, matrix metalloproteinase-9, tissue inhibitor of metalloproteinases-1, angiopoietin-2, and insulin-like growth factor binding protein-2, which are not used much in routine examinations but can be used to predict chorioamnionitis [5,6]. Therefore, an easy, cheap, and routine early diagnostic test for chorioamnionitis is needed.

A study by Cappelletti et al. [7] determined that placental inflammation is a result of histological chorioamnionitis, which can be predicted by NLR. Studies have also shown that subclinical infection is often detected in PPROM and preterm labor. Similar to previous studies, NLR values were much higher in the study group than in the control group, a difference that was statistically significant (p=0.00).

A study by Zhu et al. [8] showed that ADAMTS4 and NLRP1, NLRP3, AIM2, and NLRC4 inflammasome levels were found to increase in chorioamnionitis but with no increase in corresponding receptor levels. In their study, PPROM was considered curable by halting the increase in inflammasomes in treatment. In a study by Ozel et al. [9], the cutoff value of NLR to predict neonatal sepsis was 5.14 (AUC=0.717, 95% confidence interval=0.610-0.824, p=0.001), as compared to 6.73 in our study. Kim et al. found that NLR had a better predictive value for placental inflammation than CRP or WBC counts [10]. In another study, the cutoff value of NLR to predict placental inflammation in different gestational weeks was similar to that in our study. In addition, the authors mentioned that normal CRP levels still carry a risk for preterm delivery; similar to the selection of cases in our study, all PPROM patients had normal CRP levels.

In our study, although PLR was not statistically significant between the PPROM and control groups (p>0.05), NLR increased significantly in premature birth compared to PLR. In a study by Toprak et al., PLR and NLR were higher in PPROM compared to the control group. PLR increased the risk of PPROM over 250-fold in one study and over 117-fold in another study [11].

Uckan et al. found a positive correlation between PPROM and PCT (platelet crit), MPV (mean platelet volume), NLR, and MLR (monocyte/lymphocyte ratio). In their ROC analysis, cutoff values of PCT >0.19, MPV >8.78, NLR >2.82, and MLR >0.24 were significantly related to increased risk of PPROM (p<0.05) [12].

As NLR may be affected in pregnant women with gestational diabetes, cholestasis, hyperemesis gravidarum, or acute appendicitis, this patient group was not included in the study. In one study, NLR was found to be significantly higher in the PPROM group than in the control group, confirming that it is a valuable and accurate biomarker for PPROM prediction [13]. In another study, NLR was more predictive in preterm births, independent of CRP or leukocyte count [14].

PLR is arguably more valuable in inflammatory and thrombotic diseases, but it may be affected in pregnant women with gestational diabetes, acute pancreatitis, preeclampsia, or PPROM. However, PLR does not change in the amount of oligohydramnios and normal amniotic fluid [15]. Furthermore, as amnion in the PPROM group did not decrease immediately in the blood taken at the first stage in our study, any differences in PLR values between the PPROM and control groups (i.e., with normal amnion) may not be found statistically significant.

This study is the first to show the association between PPROM and NLR and PLR initiating from pregnancies complicated with PPROM after the 22nd gestational week. These hematological tests may be used after PPROM occurs to predict the prognosis of the pregnancy. In the literature, the earlier the PPROM occurs, the earlier the birth occurs [16-18]. Chorioamnionitis has been reported in up to 60% of cases and is thought to be a common reason for preterm birth [19]. In our study, inflammation (i.e., possibly subclinical chorioamnionitis) was supported by the statistically significant difference of inflammatory marker NLR in the PPROM group. In a study of diagnosing subclinical chorioamnionitis in PPROM by NLR, NLR was shown to be predictive of PPROM [20]. We hope that NLR screening and detection in pregnancy will occur in future studies and that pregnant women with inflammatory signs will be flagged for potential PPROM before membrane rupture occurs. This study is limited in that it cannot be known precisely whether the inflammatory markers are high from membrane rupture or vice versa, and it does not seem possible to conduct a study to predict this prospectively. Another limitation of the study is that it was retrospective and that these hematological parameters were not evaluated in patients who did not have PPROM according to gestational weeks, and the control group had similar hematological values to the PPROM group.

## Conclusions

In this study, NLR values were higher in the PPROM group than in the control group. However, further studies are needed to improve the cutoff values of NLR and other inflammatory laboratory tests. The cutoff levels of NLR should be predicted according to the gestational weeks for PPROM in further studies.
